# Evaluation of diagnostic value of pelvic MRI in endometriosis in comparison with surgical findings: A cross-sectional study

**DOI:** 10.18502/ijrm.v22i1.15242

**Published:** 2024-02-23

**Authors:** Reza Nafisi Moghadam, Fatemeh Tamizi, Seid Kazem Razavi Ratki, Amin Nafisi Moghadam, Atiyeh Javaheri, Nasim Namiranian

**Affiliations:** ^1^Department of Radiology, Faculty of Medicine, Shahid Sadoughi University of Medical Sciences, Yazd, Iran.; ^2^Faculty of Medicine, Aliebnabitaleb Azad University, Yazd, Iran.; ^3^Department of Obstetrics and Gynecology, Shahid Sadoughi Hospital, Shahid Sadoughi University of Medical Sciences, Yazd, Iran.; ^4^Diabetes Research Center, Shahid Sadoughi University of Medical Sciences, Yazd, Iran.

**Keywords:** Deep infiltrating endometriosis, Magnetic resonance imaging, Diagnosis, Laparoscopy, Sensitivity, Specificity.

## Abstract

**Background:**

Endometriosis is a multifocal gynecologic disorder during the fertility period in women. Magnetic resonance imaging (MRI) is an important diagnostic modality for this disease and can either be used alone or along with transvaginal ultrasonography.

**Objective:**

This study aims to compare the accuracy of pelvis MRI in pelvic deep endometriosis with laparoscopic findings in women referred to Shahid Sadoughi hospital in one year.

**Materials and Methods:**

This cross-sectional study was conducted on 40 women suspicious of endometriosis who referred to Shahid Sadoughi hospital, Yazd, Iran from November 2020-2021. Based on clinical findings and history, participants were referred to the imaging center for pelvic MRI. Finally, the results of MRI and diagnostic laparoscopy were compared with pathologic findings.

**Results:**

The sensitivity and specificity of MRI for pelvic endometriosis were 94.8% and 20%, respectively. Also, the positive predictive value and negative predictive value of MRI were 90.2% and 33.3%, respectively.

**Conclusion:**

Laparoscopy is still the gold standard of endometriosis diagnosis, but MRI with susceptibility-weighted imaging sequence is the best noninvasive diagnostic method.

## 1. Introduction

Endometriosis is defined as ectopic endometrial tissue including glands and stromal tissue outside the uterus (1, 2). The incidence of endometriosis varies, but the prevalence of this disease in fertile women is reported 10-15%. The most common symptoms are periodic pelvic pain and infertility (1, 3). Endometriosis is a multifocal gynecologic disorder, and may take up to 10 yr to be diagnosed. Therefore, it can have social and psychological difficulties, and it comes a lot to the health care system (4-6). There can be 3 forms of endometriosis including ovarian, peritoneal, and deep infiltrating endometriosis (DIE). DIE is one of the most important chronic pelvic pain in women and often leads to surgery. Diagnosing, staging, and treatment management is challenging. Although transvaginal ultrasonography (TVS) is considered to be the first noninvasive diagnostic method, laparoscopy is the minimally invasive method as the gold standard (7-9).

Magnetic resonance imaging (MRI) is a noninvasive and supplementary method that can detect endometrial lesions in the pelvic (7, 10). Since surgery is the best treatment for endometriosis, detection and localizing of endometriosis lesions (foci) is very important, but detecting the severity of DIE by physical examination and laparoscopy is difficult. The evaluation of DIE in occult spaces and subperitoneal areas is limited by pelvic adhesion (7, 11). MRI is an appropriate imaging modality because it provides high spatial resolution, a large field of view, and multi-planar imaging and tissue differentiation. The sensitivity and accuracy of MRI in DIE are 84% and 85%, respectively (12). However, there are some limitations in the detection of endometriosis. For instance, the diagnosis of intestinal DIE is difficult due to motion artifacts or retroflection uterus making the detection of endometriosis in uterosacral ligaments difficult.

Also, recto vaginal septal lesions are mostly seen as nodules or infiltrative masses that are mainly composed of fibrotic tissues with a few hemorrhagic foci, so they are demonstrated hypo signals in T1 and T2 weighted images in MRI. Therefore, normal fibrotic tissue in the rectovaginal septal may lead to a false positive predictive value (PPV) (13). According to the evidence, the diagnostic accuracy of MRI in pelvic endometriosis is significant, though its accuracy is variable in different parts of the pelvic (14). MRI is a valuable method for detecting endometriosis with or without TVS (15). It is important that despite all the benefits of MRI, TVS is the first choice in diagnostic methods. Although in the detection of small focus ( < 1/5 cm) in the uterosacral ligament and bladder, TVS is found to be more effective than MRI, and its accuracy for deep pelvic and superficial peritoneal lesions is not clear (12, 16).

This study aims to compare the accuracy of MRI with laparoscopic findings in deep pelvic endometriosis at Shahid Sadoughi hospital, Yazd, Iran.

## 2. Materials and Methods

This diagnostic cross-sectional study was conducted on 40 women suspicious of DIE who referred to the gynecology clinic of Shahid Sadoughi hospital, Yazd, Iran from November 2020-2021.

The inclusion criteria were ≥ 20 yr women suspicious of endometriosis who did not have contraindications for MRI. Those who visited the gynecologic clinic and were diagnosed with DIE endometriosis after checking their history, physical examination, and vaginal ultrasonography by a gynecologist were referred to the imaging center for MRI. The women were selected based on endometriosis criteria (17) that included infertility history, endometriosis surgery, dysmenorrhea, deep dyspareunia, periodic painful deification, dysuria, and asthenia or were defective with lesions in the posterior vaginal fornix, vaginal infiltration or nodules, lesion in the pouch of Douglas in vaginal and rectal examination (18). Excluding criteria included a cochlear implant, pacemaker, and claustrophobia.

All women underwent diagnosis MRI before performing the laparoscopy. Images in this study were acquired on a 1.5-T imager (Avanto; Siemens, Erlangen, Germany). Our standard imaging protocol is detailed and includes an axial dark fluid inversion-recovery T1-weighted sequence; axial and sagittal fat-suppressed fast spin-echo T1-weighted sequences; and axial, oblique coronal, and sagittal T2-weighted sequences.

In this study, contrast media was not used because there is no difference between inflammatory lesions and endometriosis foci in post-contrast images. 2 radiologists interpreted these images. They were experienced separately and the findings were rewarded. In addition, women were examined by laparoscopic surgery by a gynecologist who was blind to the results of MRI.

Pathological findings and MRI images were collected in a data sheet designed by the researchers and compared.

### Sample size

The sample size of 40 women was determined according to Thomeer et al. study and interval coefficient of 95% (16). The α was 0.05 and the β was 20%. Also 15% attrition rate was considered.

### Ethical considerations

The proposal was confirmed by the Ethics Committee of Shahid Sadoughi University of Medical Sciences, Yazd, Iran (Code: IR.SSU.MEDICINE.REC.1398.303). The researchers respected the Helsinki Declaration throughout the process. Written consent forms were obtained from all participants, and they were assumed that their data would be considered confidential and would only be used for research purposes. It was also guaranteed that this research would have no effect on their treatment process and would not cost any expenses.

### Statistical analysis

Data were analyzed using SPSS (Statistical Package for the Social Sciences, version 22.0, SPSS Inc., Chicago, Illinois, USA) to calculate the mean value standard deviation and relative frequency. The sensitivity and specificity of laparoscopic and MRI findings were calculated.

## 3. Results

This study involved 40 women suspected of having DIE (aged 20-67 yr, with a mean age of 37.75 ± 8.34 yr). Among the participants, 16 (40%) had a history of infertility, 9 (22.5%) had undergone endometriosis surgery, 18 (45%) reported dyspareunia, 31 (77.5%) reported dysmenorrhea, and 2 (5%) reported dysuria (each woman had one or more of these signs and symptoms).

Laparoscopy and MRI findings regarding adhesions and fibrosis, endometrioma, uterosacral ligaments, rectouterine pouch, rectovaginal pouch, and GI tract lesions were compared (Table I). The sensitivity and specificity (95% CI), PPV, and negative predictive value (NPV) were also reported.

According to laparoscopy as a gold standard method in endometriosis detection, the sensitivity and specificity of MRI in endometriosis disease were 94.8% (9.1-99.8%) and 20% (11-67%), respectively. PPV and NPV of MRI were 90.2% and 33.3%, respectively.

**Table 1:** The compassion of laparoscopic and MRI findings


**Laparoscopy**/**MRI**		Positive*	Negative*	Sensitivity**	Specificity**
**Lesions of adhesion and fibrosis**					
	**Positive**	13 (32.5)	0 (0)	
	**Negative**	19 (47.5)	8 (20)	40 (29.7-70.3)	100 (63.3-100%)
**Endometrioma**					
	**Positive**	34 (85)	1 (2.5)	
	**Negative**	1 (2.5)	4 (10)	97.1 (85.08-99.9)	80 (38.36-99.4)
**Lesions of uterosacral ligaments**					
	**Positive**	15 (37.5)	8 (20)	
	**Negative**	6 (15)	11 (27.5)	71.4 (47.8-88.7)	57.8 (33.7-79.8)
**Lesions of rectouterine pouch**					
	**Positive**	4 (10)	4 (10)	
	**Negative**	8 (20)	24 (60)	33.3 (15.9-84.4)	85.7 (56.6-88.5)
**Lesions of rectovaginal pouch**					
	**Positive**	37 (92.5)	3 (7.5)	
	**Negative**	0 (0)	0 (0)	100 (91.1-100)	90 (76.7-100)
**Lesions of GI tract**					
	**Positive**	1 (2.5)	2 (5)	
	**Negative**	19 (47.5)	18 (45)	5 (0-23.4)	90 (87.7-100)
*Data presented as n (%). **Data presented as percentage (95% CI). MRI: Magnetic resonance imaging, GI: Gastrointestinal					

## 4. Discussion

In this study, 40 women suspected of endometriosis were investigated by MRI and laparoscopy. Generally, pelvic MRI in endometriosis was efficient with a sensitivity of 94.8%. However, the specificity of findings is about 20%. The sensitivity and specificity of endometriosis are different depending on the anatomic location and size of the lesions. The difference in MRI accuracy in various anatomic locations is also reported earlier expected that the detection of deep pelvic endometriosis and adhesion bands would be more accurate by laparoscopy compared to MRI (14). The detection of endometrioma is highly accurate by MRI and laparoscopy because the appearance of these lesions is large and cystic. Our study showed the detection of uterosacral ligament lesions. MRI can detect utero sacral ligament lesions as efficiently as laparoscopy, but it is not efficient enough in the detection of the GI tract.

In general, PPV is 90.2% and NPV is 33.3% for all detected lesions by MRI. In a study out of 363 cases suspicious of endometriosis, 89 patients were operated, and 79 of them were investigated on the history, physical examination, ultrasonography, and MRI. Their study showed that if MRI is added to other methods for diagnosing endometriosis, sensitivity will decrease from 93.7-85.9, and specificity will increase from 55.6-62.5. Thus, the authors concluded that MRI does not significantly help diagnose endometriosis (19).

In our study, MRI was an efficient method in detection of endometrioma while it was less efficient in the detection of deep endometriosis, they have used MRI in endometriosis depending on the kind of lesions. Our findings demonstrate that the PPV and sensitivity of MRI in detecting the most of lesions are acceptable, but the specificity and NPV of these lesions are low. So, in cases suspicious of endometriosis, MRI can be recommended as a non-interventional method for diagnosis. If lesions are detected by MRI, laparoscopic surgery is indicated. MRI helps reduce unnecessary surgery to some extent which decreases medical costs for patients.

In another study, 74 women suspected to have DIE were examined by ultrasonography, urethrography, barium enema, and MRI to evaluate pelvic lesions. The image findings were compared with laparoscopic findings as the gold standard method (20). It increased the diagnostic value percentage from 2.7-6.8 using MRI which is compatible with our study. In a similar study, 42 women were diagnosed with endometriosis by pathologic samples of which 28 cases were detected with MRI. MRI was suggested for 5 suspicious endometriosis and 9 cases were reported normal. As a result, the sensitivity of MRI was 69% and the specificity was 75% (21). This difference can be related to the experience of the radiologist. It was shown that the experience of the radiologist and their relationship with the gynecologist is of great importance in the diagnosis of endometriosis (22). In our study, the radiologist was experienced enough to report pelvic MRI.

In another study, 74 patients were examined and 10 of them were operated. In MRI, lesions were detected in 52% of patients in T1w MRI and were increased to 81% in SW images in MRI. In their study, increased accuracy of susceptibility weighted imaging (SWI) especially, in rectovaginal and uterosacral ligaments were approved. So SWI was suggested as a complementary sequence in MRI (23). As a result, SWI can be added to conventional sequences (T1w and T2w) to improve the diagnosis.

## 5. Conclusion

Non-interventional diagnosis of endometriosis is a big challenge for gynecologists and radiologists. MRI can be used as an efficient method for the detection of endometrial lesions and can decrease laparoscopic surgery. It is recommended that similar feature studies are conducted on larger samples and the use of SWI sequences in MRI imaging.

##  Data availability

Data supporting the findings of this study are available upon reasonable request from the corresponding author.

##  Author contributions

RNM, FT and AJ designed the study and conducted the research. NN, SKRR and FT monitored, evaluated, and analyzed the result of the study. ANM drafted the manuscript. RNM, FT, SKRR, ANM, AJ, NN reviewed the article. All authors approved the final manuscript and take responsibility for the integrity of the data.

##  Conflict of Interest

The authors declare that there is no conflict of interest.

## References

[bib1] McGregor GR, Vanos JK (2018). Heat: A primer for public health researchers. Public Health.

[bib2] Kastelic JP, Rizzoto GJBr (2021). Thermoregulation of the testes.

[bib3] Abd El-Emam MM, Ray MN, Ozono M, Kogure K (2023). Heat stress disrupts spermatogenesis via modulation of sperm-specific calcium channels in rats. J Therm Biol.

[bib4] Durairajanayagam D, Agarwal A, Ong C (2015). Causes, effects and molecular mechanisms of testicular heat stress. Reprod Biomed Online.

[bib5] Mäkelä J-A, Koskenniemi JJ, Virtanen HE, Toppari J (2019). Testis development. Endocr Rev.

[bib6] O’Donnell L, Smith LB, Rebourcet D (2022). Sertoli cells as key drivers of testis function. Semin Cell Dev Biol.

[bib7] Arisha AH, Ahmed MM, Kamel MA, Attia YA, Hussein MMA (2019). Morin ameliorates the testicular apoptosis, oxidative stress, and impact on blood–testis barrier induced by photo-extracellularly synthesized silver nanoparticles. Environ Sci Pollut Res Int.

[bib8] Hu Y, Hu H, Yin L, Wang L, Luo K, Luo N, et al (2023). Arachidonic acid impairs the function of the blood-testis barrier via triggering mitochondrial complex-ROS-P38 MAPK axis in hyperthermal Sertoli cells. Ecotoxicol Environ Saf.

[bib9] Mruk DD, Cheng CY (2015). The mammalian blood-testis barrier: Its biology and regulation. Endocr Rev.

[bib10] Chen S-R, Liu Y-X

[bib11] Hai Y, Hou J, Liu Y, Liu Y, Yang H, Li Z, et al (2014). The roles and regulation of Sertoli cells in fate determinations of spermatogonial stem cells and spermatogenesis. Semin Cell Dev Biol.

[bib12] Hajian Monfared M, Minaee B, Rastegar T, Khrazinejad E, Barbarestani M (2016). Sertoli cell condition medium can induce germ like cells from bone marrow derived mesenchymal stem cells. Iran J Basic Med Sci.

[bib13] Panahi S, Karamian A, Sajadi E, Aliaghaei A, Nazarian H, Abdi Sh, et al (2020). Sertoli cell-conditioned medium restores spermatogenesis in azoospermic mouse testis. Cell Tissue Res.

[bib14] Afshar A, Aliaghaei A, Nazarian H, Abbaszadeh H-A, Naserzadeh P, Fathabadi FF, et al (2021). Curcumin-loaded iron particle improvement of spermatogenesis in azoospermic mouse induced by long-term scrotal hyperthermia. Reprod Sci.

[bib15] Ziaeipour S, Piryaei A, Aliaghaei A, Nazarian H, Naserzadeh P, Ebrahimi V, et al (2021). Chronic scrotal hyperthermia induces azoospermia and severe damage to testicular tissue in mice. Acta Histochem.

[bib16] Ayoubi M, Naserzadeh P, Hashemi MT, Rostami MR, Tamjid E, Tavakoli MM, et al (2017). Biochemical mechanisms of dose-dependent cytotoxicity and ROS-mediated apoptosis induced by lead sulfide/graphene oxide quantum dots for potential bioimaging applications. Sci Rep.

[bib17] Oliveira PF, Alves MG

[bib18] Parekh PA, Garcia TX, Hofmann M-C

[bib19] Peng YJ, Tang XT, Shu HS, Dong W, Shao H, Zhou BO (2023). Sertoli cells are the source of stem cell factor for spermatogenesis. Development.

[bib20] Meroni SB, Galardo MN, Rindone G, Gorga A, Riera MF, Cigorraga SB (2019). Molecular mechanisms and signaling pathways involved in sertoli cell proliferation. Front Endocrinol.

[bib21] Muratori M, Tamburrino L, Marchiani S, Cambi M, Olivito B, Azzari C, et al (2015). Investigation on the origin of sperm DNA fragmentation: Role of apoptosis, immaturity and oxidative stress. Mol Med.

[bib22] Fu L, Yuen KCJ, Tint AN, Hoffman AR, Bongso AT, Lee KO (2021). Association of decreased sperm motility and increased seminal plasma IGF-I, IGF-II, IGFBP-2, and PSA levels in infertile men. Endocrine.

[bib23] Ghafouri-Fard S, Shoorei H, Mohaqiq M, Raza SHA, Taheri M (2021). The role of different compounds on the integrity of blood-testis barrier: A concise review based on in vitro and in vivo studies. Gene.

[bib24] Su L, Wang Z, Xie S, Hu D, Cheng YC, Mruk DD, et al (2020). Testin regulates the blood‐testis barrier via disturbing occludin/ZO‐1 association and actin organization. J Cell Physiol.

[bib25] Piprek RP, Kloc M, Mizia P, Kubiak JZ (2020). The central role of cadherins in gonad development, reproduction, and fertility. Int J Mol Sci.

[bib26] Zhang DC, Chen R, Cai YH, Wang JJ, Yin C, Zou K (2020). Hyperactive reactive oxygen species impair function of porcine Sertoli cells via suppression of surface protein ITGB1 and connexin-43. Zool Res.

[bib27] Yang W-R, Li B-B, Hu Y, Zhang L, Wang X-Z (2020). Oxidative stress mediates heat-induced changes of tight junction proteins in porcine sertoli cells via inhibiting CaMKKβ-AMPK pathway. Theriogenology.

[bib28] Hofmann M-C, McBeath E (2022). Sertoli cell-germ cell interactions within the niche: Paracrine and juxtacrine molecular communications. Front Endocrinol.

[bib29] Lv D, Zhao M, Ni J, Liu W, Ren Y, Zhu D, et al (2023). NGF regulates sertoli cell growth and prevents LPS-induced junction protein damage via PI3K/AKT/NFκB signaling. Theriogenology.

